# Filamin B (*FLNB*)-Related Spondylocarpotarsal Synostosis Syndrome: Systematic Literature Review and Novel Case Report

**DOI:** 10.3390/genes17091054

**Published:** 2026-08-31

**Authors:** Chiara Gobbetto, Sofia Passarella, Thomas Zoller, Ruggero Lanzafame, Rossella Gaudino, Irene Ambrosetti, Angelo Pietrobelli, Franco Antoniazzi

**Affiliations:** 1 Paediatric Unit, Department of Surgical Science, Dentistry, Gynecology and Paediatrics, Azienda Ospedaliera Universitaria Integrata Verona, P.le Stefani 1, 37135 Verona, Italy; 2 Division of Paediatrics, Department of Medicine (DMED), University of Udine, 33100 Udine, Italy; 3 Clinical Genetics Unit, Azienda Ospedaliera Universitaria Integrata Verona, 37134 Verona, Italy; 4 Pennington Biomedical Research Center, Baton Rouge, LA 70808, USA

**Keywords:** spondylocarpotarsal synostosis syndrome, FLNB, filamin B, vertebral fusion, carpal synostosis, skeletal dysplasia, short stature, case report, review

## Abstract

Spondylocarpotarsal synostosis syndrome (SCT) is a rare autosomal recessive skeletal dysplasia caused by biallelic loss-of-function variants in *FLNB*, characterized by disproportionate short stature, progressive vertebral fusion, scoliosis, and carpal (typically capitate–hamate) synostosis, classically without rib anomalies. We performed a systematic literature search of PubMed/MEDLINE and Scopus, supplemented by citation searching, following the PRISMA 2020 statement, to identify all reports providing original, molecularly confirmed *FLNB*-related SCT patient data. Eleven eligible publications, spanning Taiwanese, Argentinian, Pakistani, Italian, German and Indian cohorts, were included in a qualitative synthesis together with a novel case. The novel patient, an adopted child of Indian origin, presented with severe disproportionate short stature, multisegmental vertebral fusion and posterior arch clefts extending from the cervical to the sacral spine, coccygeal dysgenesis, capitate–hamate fusion, and global developmental delay; targeted exome sequencing identified a novel homozygous frameshift *FLNB* variant. Integration of this case with the reviewed literature confirms disproportionate short stature, contiguous vertebral fusion, and carpal synostosis as near-universal core features, while tarsal fusion, hearing loss, and facial dysmorphism are variably present. The extent and multisegmental pattern of axial involvement in our patient appear more severe than in most previously reported cases, broadening the recognized phenotypic spectrum of *FLNB*-related SCT and reinforcing the need for structured, multidisciplinary long-term follow-up.

## 1. Introduction

Spondylocarpotarsal synostosis syndrome (SCT) was first delineated as a distinct clinical entity by Jones et al. in 1973 [1] and subsequently named by Langer et al. in 1994 [2]. The latter proposed an autosomal recessive mode of inheritance based on sibling recurrence in non-consanguineous and consanguineous families. The condition is defined by the combination of disproportionate short stature with a short trunk, progressive fusion of vertebral bodies and posterior elements (most often contiguous thoracic segments), scoliosis and/or lordosis, and synostosis of carpal bones—most characteristically the capitate and hamate—with variable tarsal involvement [3,4,5,6,7,8]. Unlike spondylocostal dysostosis and related segmentation disorders, SCT is classically distinguished by the absence of rib anomalies, although exceptions have since been documented [9]. In 2004, Krakow et al. localized the disease-causing locus to chromosome 3p14.3 and subsequently identified homozygous or compound heterozygous nonsense variants in the *FLNB* gene, which encodes the cytoskeletal protein filamin B, in four families with autosomal recessive SCT [10].

Filamin B is expressed in the chondrocytes of the cartilage growth plate and in developing vertebral bodies, consistent with its central role in endochondral ossification, vertebral segmentation, and joint formation [11,12].

This article aims to summarize current knowledge of the disease by describing patients with molecularly confirmed *FLNB*-related SCT and to extend it with a novel case report.

## 2. Materials and Methods

### 2.1. Novel Case Report-Genetic Analysis

To enrich the existing body of literature, we present detailed clinical, radiographic, and genetic information from our own case. Clinical data were collected through routine clinical evaluation performed at our institution, including physical examination, radiographic assessment, and laboratory investigations. Diagnostic work-up included skeletal radiographs and genetic testing. Genetic analysis was performed as a clinical diagnostic service by the SOSD Genetica Medica e Citogenetica Molecolare, Azienda Ospedaliero Universitaria Meyer IRCCS, Florence, Italy, using the KAPA HyperExome liquid capture kit (Roche NimbleGen, Basel, Switzerland) with paired-end 150 bp sequencing on the Illumina NovaSeq 6000 platform (San Diego, California, USA). Genomic DNA was extracted from peripheral blood leukocytes. Reads were aligned to the GRCh37 reference genome using BWA v.0.7.17; variants were called with GATK HaplotypeCaller v.4.2.6.1 and annotated using ANNOVAR (v. 20200607), with analysis focused on an in silico gene panel for skeletal dysplasias. The mean sequencing depth was 133×, with 98.5% of targeted bases covered at ≥30×. Variants were interpreted against population databases, including gnomAD v4.0.0 (excluding those with allele frequency ≥ 1). Variant prioritization was based on several criteria, including inheritance pattern, variant type, previously reported disease-associated variants in ClinVar and HGMD, and predictions from multiple in silico tools such as REVEL, MetaRNN, CADD, PolyPhen-2, SIFT, MutationTaster, MutationAssessor, FATHMM, FATHMM-MKL, SpliceAI, and ADA score. Variant classification followed ACMG/AMP guidelines. The homozygous frameshift variant c.[7332dup]; [7332dup] (NM_001457.4; p.[Met2445HisfsTer5]; [Met2445HisfsTer5]) was identified and classified as likely pathogenic (PVS1, PM2). Independent confirmation by Sanger sequencing was performed as part of the clinical diagnostic workflow. Segregation analysis could not be performed, as biological parental samples were unavailable owing to the patient’s adoption status; this limitation is explicitly stated here to allow appropriate interpretation of the molecular findings. Written informed consent for the publication of clinical data and images was provided by the patient’s legal guardians, in accordance with the Declaration of Helsinki.

According to common international and national practice, case reports are considered descriptive medical observations rather than clinical research. For this reason, no formal approval from an Ethics Committee or Institutional Review Board was required or sought.

### 2.2. Systematic Review Protocol and Reporting Standard

This methodology was conducted and is reported in accordance with the Preferred Reporting Items for Systematic Reviews and Meta-Analyses (PRISMA) 2020 statement. The objective of the literature search was to identify all published reports providing original, patient-level clinical, radiological, and/or molecular data on individuals affected by spondylocarpotarsal synostosis syndrome (SCT) with a molecularly confirmed pathogenic/likely pathogenic variant in *FLNB*.

### 2.3. Information Sources and Search Strategy

Two bibliographic databases were searched on 24 June 2026: PubMed/MEDLINE and Scopus. No date or language restrictions were applied at the search stage.

PubMed search string (Title/Abstract fields):

((Spondylocarpotarsal synostosis syndrome[Title/Abstract]) OR (SCT[Title/Abstract])) AND (FLNB[Title/Abstract])

Scopus search string (Article title, Abstract, Keywords fields):

(“spondylocarpotarsal synostosis” OR SCT) AND FLNB

The PubMed search returned 24 records (exported as a formatted abstract/citation set). The Scopus search returned 25 records (exported as a structured CSV including title, authors, year, source, abstract, DOI, and document type). Both export files were used as the primary information sources for this review.

### 2.4. Supplementary Identification via Citation Searching

Both database search strings require the disease descriptor—“spondylocarpotarsal synostosis syndrome” or the acronym “SCT”—to appear in the title or abstract. This criterion, while specific and reproducible, does not retrieve the original report that first established the gene–disease relationship underpinning this entire review: Krakow et al., Nature Genetics 2004 (PMID 14991055), “Mutations in the gene encoding filamin B disrupt vertebral segmentation, joint formation and skeletogenesis” [10]. That report’s title and abstract describe the phenotype only as “vertebral segmentation, joint formation and skeletogenesis” defects; the term “spondylocarpotarsal” does not appear, because standardized naming of the syndrome post-dates this publication. The article was nonetheless identified because it is cited as the original molecular description of *FLNB*-related SCT by every other report retrieved through the database search.

Because broadening the database strings with non-specific terms such as “vertebral segmentation” would have introduced a large volume of irrelevant results from unrelated fields (e.g., spinal imaging and radiological segmentation-algorithm literature), the database strings were kept narrow and reproducible, and this single additional record was instead incorporated through citation searching of the reference lists of the reports retrieved by the database search—corresponding to the “identification via other methods” pathway of the PRISMA 2020 flow diagram. No other additional records were identified through citation searching.

### 2.5. Results of the Search and Study Selection

**Figure 1:**
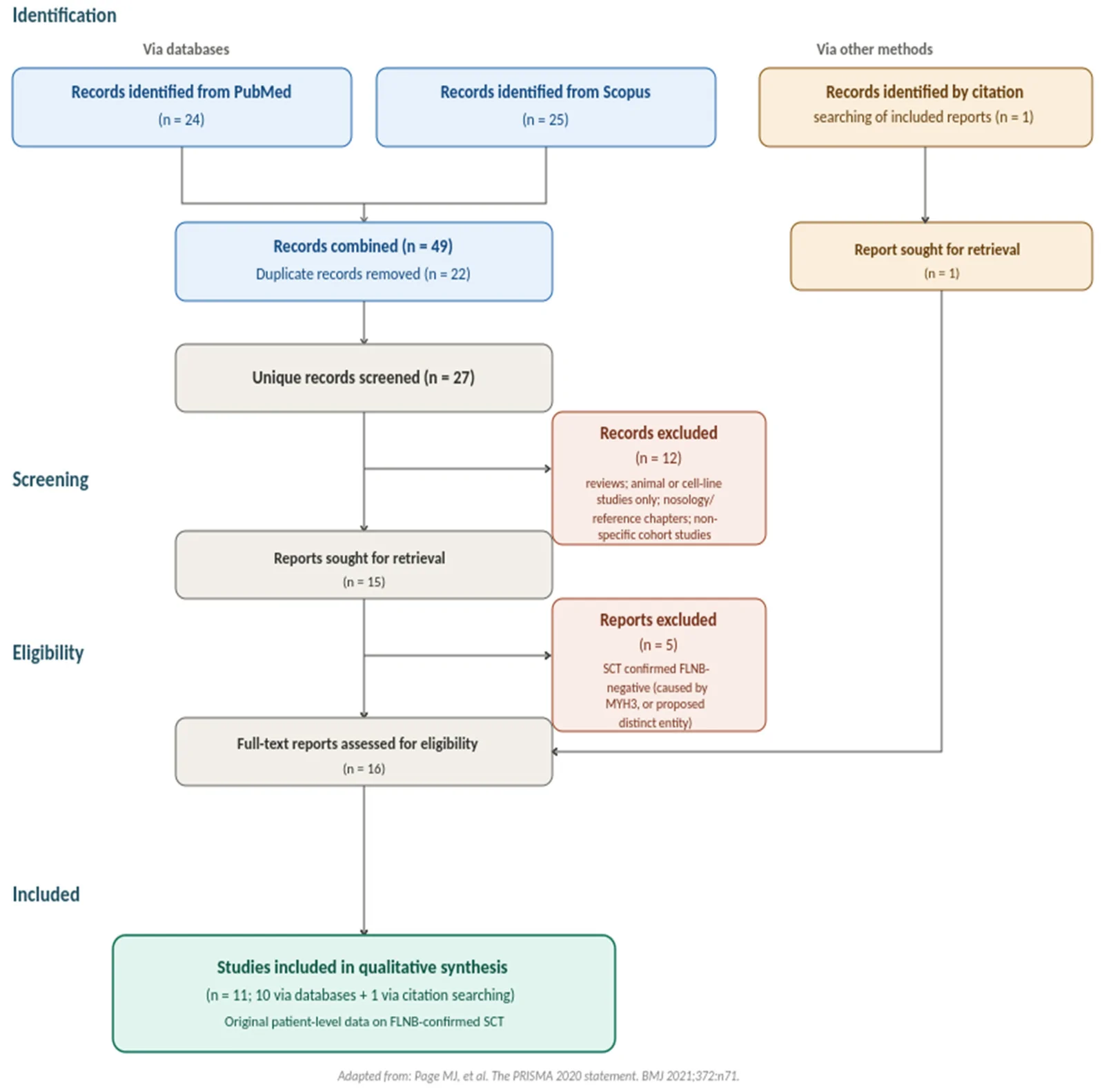
PRISMA 2020 flow diagram of the identification, screening, eligibility, and inclusion of studies reporting original *FLNB*-confirmed SCT patient data.

The combined search of PubMed (*n* = 24) and Scopus (*n* = 25) yielded 49 database records. After removal of 22 duplicate records indexed in both databases, 27 unique database records remained for title/abstract screening. Of these, 12 were excluded at the screening stage as clearly ineligible by design (narrative reviews, animal- or cell-line-only mechanistic studies, reference/nosology documents, or non-specific cohort studies), leaving 15 database reports sought for retrieval. One additional record (Krakow et al. 2004, PMID 14991055) [10] was identified through citation searching of the reference lists of the database-derived reports (Section 2.3) and was likewise sought for retrieval. In total, 16 reports were assessed in full text against the eligibility criteria; 5 were excluded because *FLNB* was not confirmed as the cause of the reported SCT phenotype (4 attributable to *MYH3*, 1 proposed as a distinct, non-*FLNB* entity). Eleven studies—10 identified via the database search and 1 via citation searching—met all eligibility criteria and were included in the qualitative synthesis. The study selection process is summarized in Figure 1.

## 3. Results

### 3.1. Novel Case Report

We report the case of a female child of Indian origin adopted by an Italian couple; information regarding pregnancy, family history, parental consanguinity and perinatal history was unavailable. According to the original medical documentation, she was born preterm, with a birth weight of 1560 g, and required neonatal intensive care admission with ventilatory support (gestational age not available).

She spent her first months of life in an Indian pediatric institute, where she was fed mainly milk and received limited developmental stimulation.

When she arrived in Italy at 15 months of age, the child presented with severe short stature (−5.19 Z-score, CDC growth charts) and global developmental delay and was admitted to a pediatric rehabilitation center to investigate the underlying etiology. She was subsequently followed up by several different hospitals, where she received multidisciplinary investigations.

**Figure 2:**
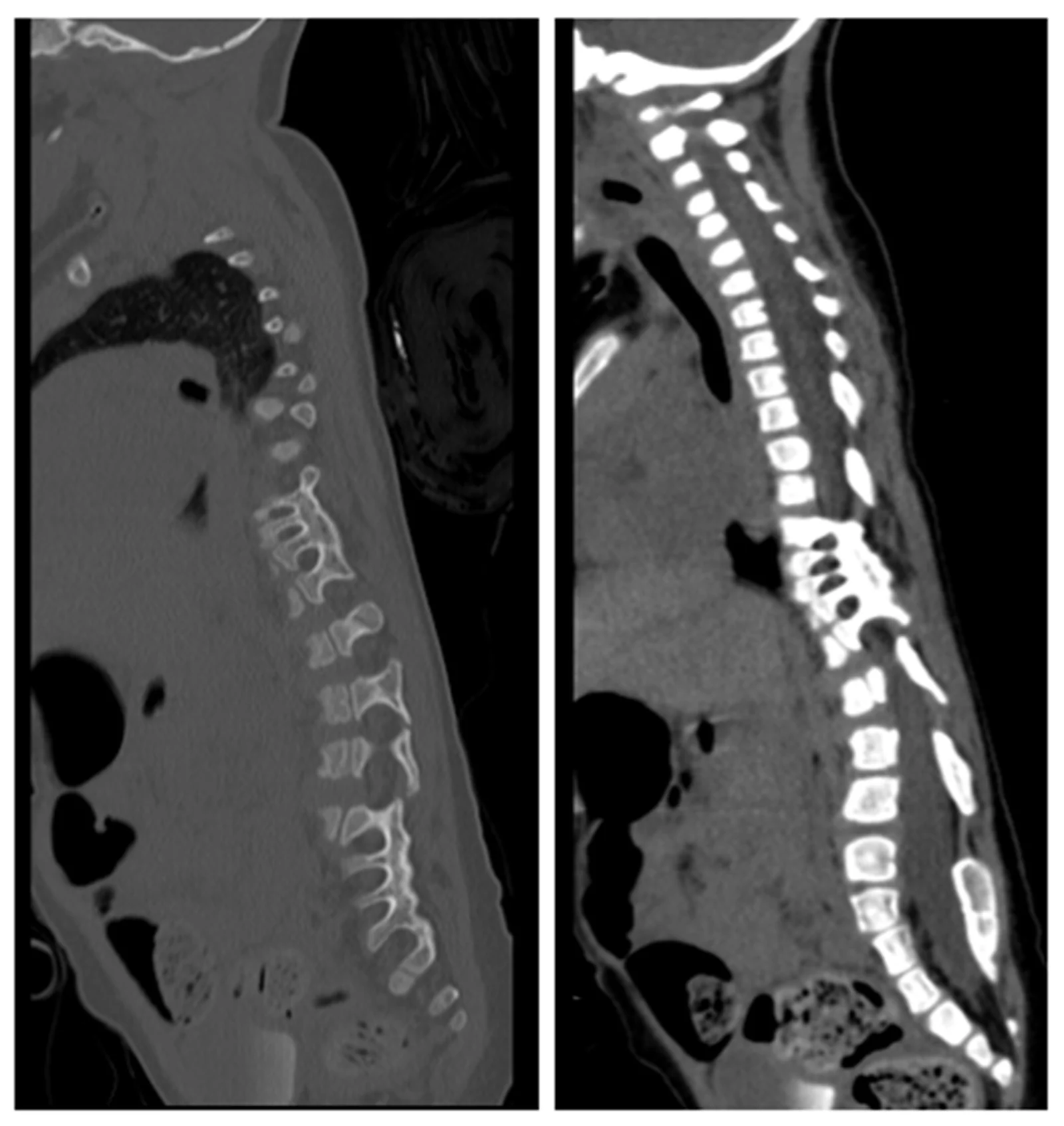
Spinal MRI (**left**) and CT scan (**right**) of the index patient at 18 months of age. MRI demonstrates multisegmental fusion of posterior vertebral elements from D1 to D11 and L1 to S2, with posterior arch clefts at C1–C2, D1–D2, D5–D11 and the sacral bodies, as well as coccygeal dysgenesis. CT confirms the complex pattern of vertebral segmentation anomalies and allows detailed assessment of posterior element involvement. A left-convex scoliotic curve is present at the thoracolumbar junction with kyphotic deformity from D10 to L2.

Documents regarding investigations previously performed in India included a spine MRI at 7 months of age showing fusion of the posterior elements (including spinous processes) of D7–D9. For this reason, brain MRI and full spine MRI plus CT were repeated at 1 year and 6 months of age. Brain imaging revealed hyperintensity of the periventricular white matter, associated with reduced cerebral white matter volume and a relatively thin corpus callosum, consistent with possible periventricular leukomalacia; these findings were interpreted as more likely related to prematurity and possible perinatal hypoxic–ischemic injury. Full spine MRI and CT showed a complex pattern of axial skeletal malformation (Figure 2). A left-convex scoliotic curve was present at the thoracolumbar junction, with kyphotic deformity from D10 to L2 and straightening of the normal thoracic kyphosis. Detailed anomalies included posterior arch clefts of C1 and C2, D1–D2, D5–D11 and the sacral bodies, together with fusion of the posterior elements of C4–C5, D1–D11 and L1–S2, and coccygeal dysgenesis.

Orthopedic evaluation described an altered sagittal spinal profile without evidence of vertebral rotation or rapidly progressive scoliosis, and neurosurgical review did not indicate surgical management.

At 1 year and 6 months of age, ophthalmologic examination revealed intermittent convergent strabismus, and dental assessment showed delayed tooth eruption, incomplete deciduous dentition, and a supernumerary tooth. Metabolic screening, cardiologic evaluation, and abdominal ultrasound were unremarkable. ENT assessment documented bilateral otitis media with effusion, whereas serial audiologic investigations were normal.

A hand radiograph obtained at 22 months of age showed a bone age delayed by approximately 1 year, with an incidental suspected fusion of the capitate and hamate ossification centers. Pelvic radiographs showed preserved joint relationships and normal pelvic bone development.

In consideration of the severe short stature and radiological anomalies, the patient was first tested with array-CGH analysis, which was normal. Subsequently, next-generation sequencing (NGS) using an in silico skeletal dysplasia gene panel identified a homozygous *FLNB* variant, c.7332dup (NM_001457.4). This duplication is predicted to shift the canonical reading frame and introduce a premature stop codon (p.Met2445HisfsTer5). According to ACMG criteria, the variant was classified as likely pathogenic, based on PVS1 and PM2 evidence, for autosomal recessive spondylocarpotarsal synostosis syndrome.

The first pediatric endocrinology evaluation was conducted at 2 years and 4 months of age. She measured 70 cm in length (−5 Z-score, CDC growth charts), with a weight of 7.2 kg (−6.6 Z-score, CDC growth charts), a head circumference of 45 cm (−1.9 Z-score, CDC charts), and an arm span of 65 cm (arm span/height ratio of 0.93).

Review of medical history revealed that, before adoption, head control was not optimal; when she arrived in Italy at 16 months, rolling was possible but impaired. Crawling appeared later, and standing with anterior support and lateral stepping emerged around 25 months. The child achieved independent walking at 2 years of age.

**Figure 3:**
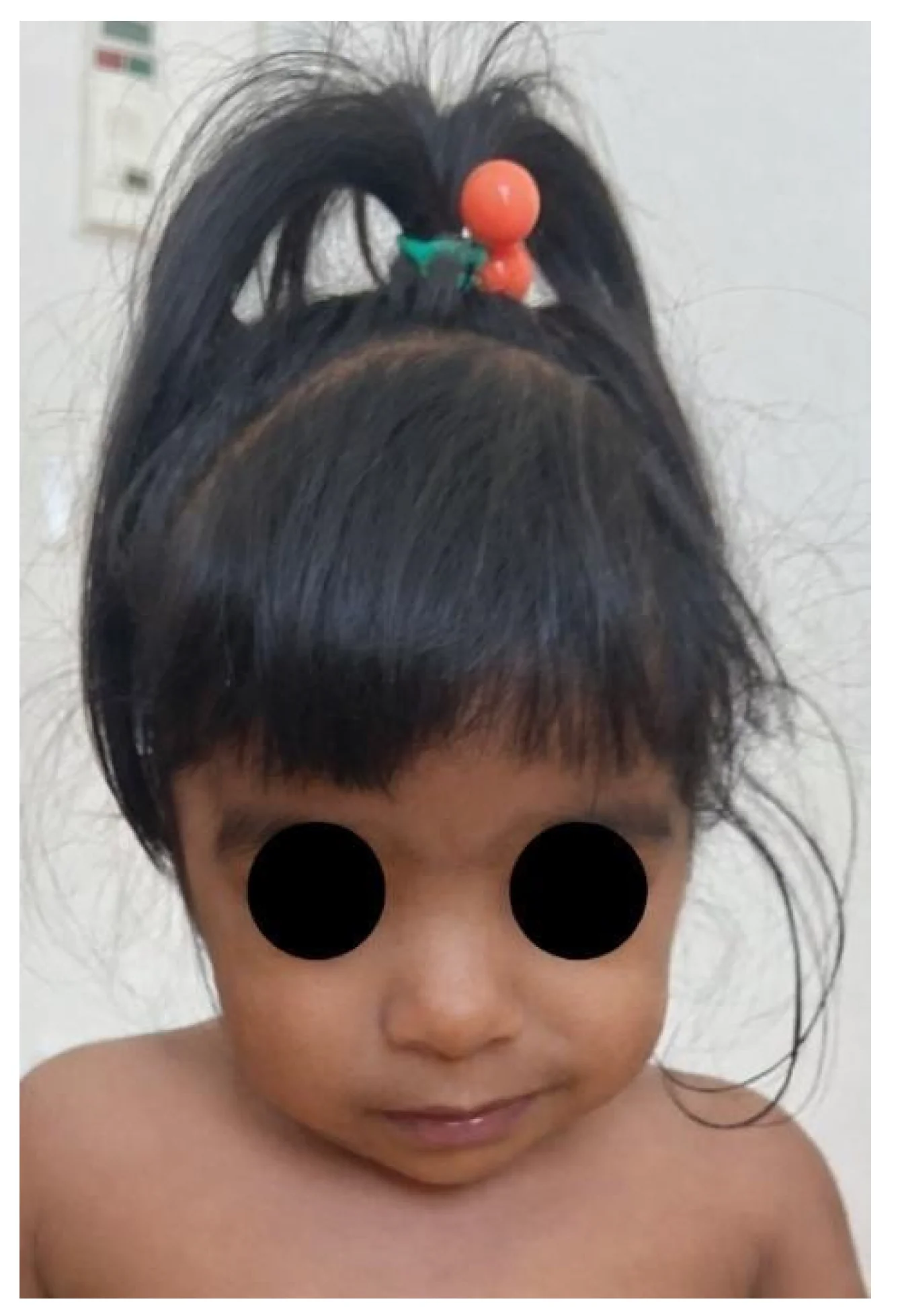
Facial appearance of the child.

On physical examination, she showed straightening of the normal thoracic kyphosis and no clinically evident scoliosis. No lower-limb length discrepancy was observed. Dysmorphic features included a triangular face with a pointed chin, a broad-based nose, a thin upper lip vermillion, a short neck, intermittent convergent strabismus, synophrys, and mild craniofacial disproportion (Figure 3). Cutaneous assessment revealed multiple café-au-lait macules of the lower limbs and trunk (for which diagnostic investigation is underway). She walked independently, with a broad-based gait, and was only babbling.

**Figure 4:**
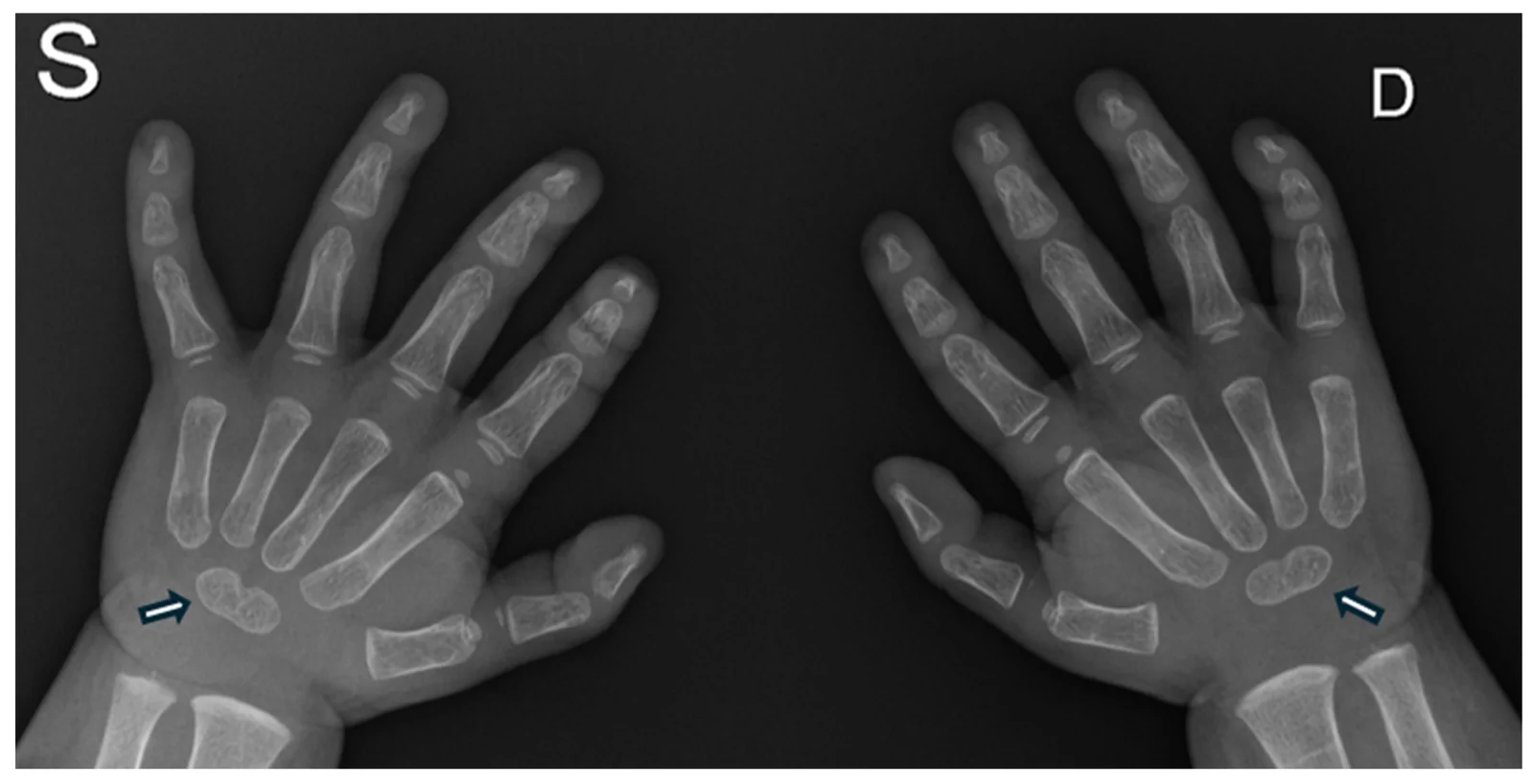
Hand radiograph at 2 years and 4 months of age demonstrating delayed bone age (assessed as approximately 9 months by the Greulich and Pyle method) and fusion of the capitate and hamate ossification centers (arrow), consistent with the carpal synostosis characteristic of *FLNB*-related SCT.

**Figure 5:**
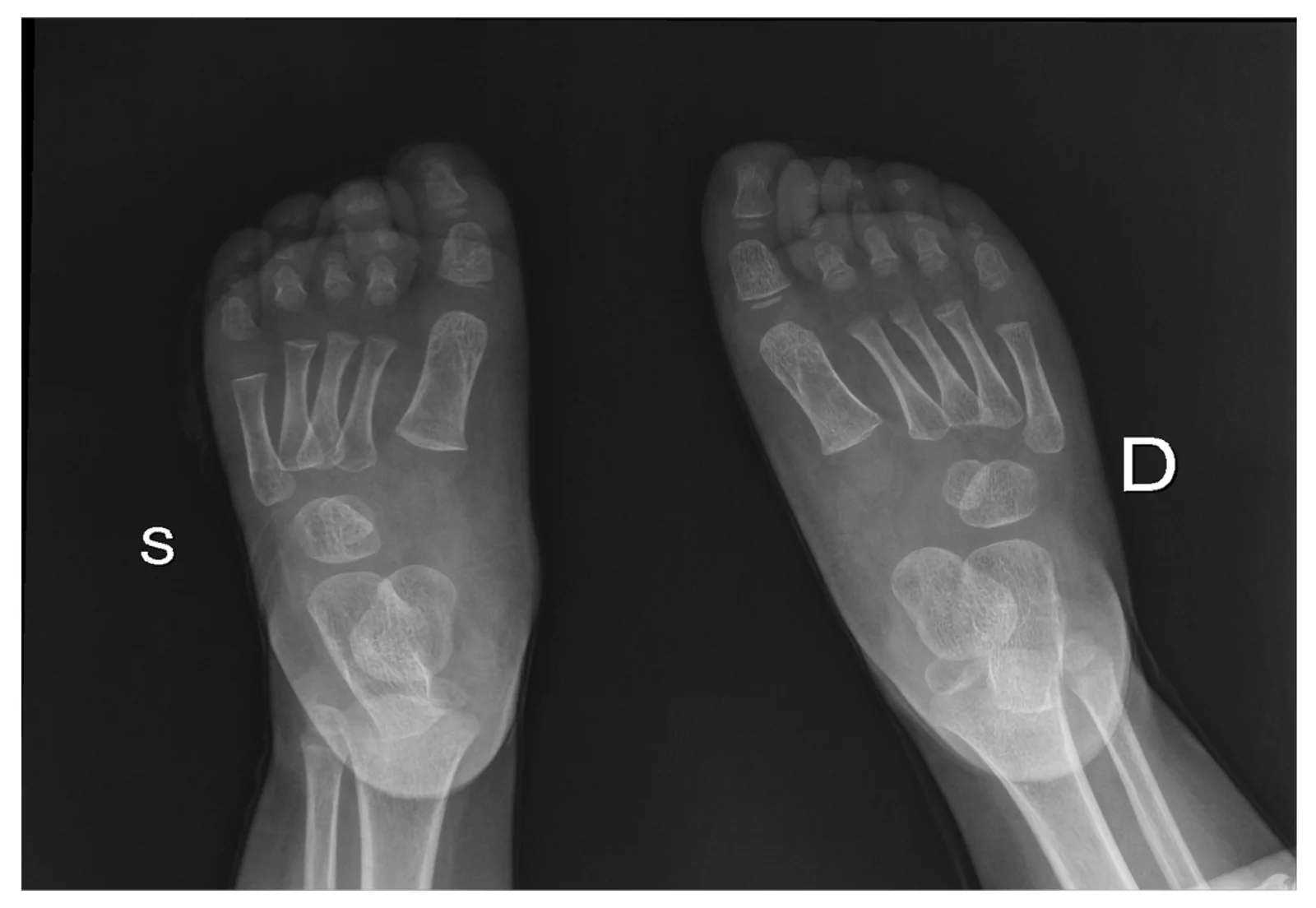
Foot radiographs demonstrating absence of the navicular ossification center.

Further investigations, including blood tests, excluded major endocrine, nutritional, hepatic, renal, electrolyte, thyroid, celiac, and hematologic abnormalities. To complete the diagnostic work-up, hand radiographs were requested and confirmed fusion of the capitate and hamate ossification centers, together with a bone age delay (bone age of 9 months at a chronological age of 2 years and 4 months) (Figure 4). Foot radiographs showed absence of the navicular bone ossification center (Figure 5); no definite tarsal synostosis was documented at this stage.

#### Proposed Follow-Up for This Patient

Although no disease-specific treatment exists, SCT requires structured multidisciplinary surveillance. Published cases suggest that long-term morbidity is mainly driven by the progression of spinal deformity and, in more severe cases, by thoracic restriction and orthopedic disability [3,8,13].

For our patient, we suggested a multidisciplinary follow-up, including:

- orthopedic and physiatric monitoring, with clinical and radiographic assessment of scoliosis, kyphosis, and sagittal profile;
- neurosurgical surveillance, particularly because of the narrow foramen magnum and complex cervical anomalies, with repeat imaging if neurologic symptoms emerge;
- endocrine and auxologic follow-up, mainly to monitor growth trajectory and nutritional status;
- audiologic reassessment, given the reported risk of conductive or sensorineural hearing loss [3,4,8];
- ophthalmologic and orthoptic follow-up, especially in view of the intermittent strabismus and reported ophthalmologic involvement in some series [3];
- neurodevelopmental and rehabilitative care, because of the global developmental delay and probable multifactorial neurologic vulnerability;
- dental surveillance, due to delayed eruption and the known association with enamel defects [5,8,13];
- respiratory evaluation when age permits, especially if axial deformity progresses and restrictive impairment becomes a concern [8];
- renal imaging, given the occasional association with renal cysts or urolithiasis [8].

### 3.2. Review of the Literature

In 2004, Krakow et al. mapped the causative gene to chromosome 3p14.3 and identified homozygous or compound heterozygous nonsense pathogenic variants in *FLNB*, encoding the cytoskeletal protein filamin B, in four families with autosomal recessive SCT. This founding observation established *FLNB* loss-of-function as the principal molecular cause of the syndrome and placed SCT within a broader spectrum of *FLNB*-related skeletal dysplasias [8,10,14,15].

Since 2004, a growing series of case reports and small case series have expanded both the clinical phenotype and the mutational spectrum of *FLNB*-related SCT. This review consolidates findings from eleven publications—spanning Taiwanese, Argentinian, Pakistani, Italian, German, and Indian cohorts—to provide an integrated picture of the clinical, radiological, and genetic features reported to date, and to summarize the variant spectrum across the *FLNB* gene. From the previously published case reports, we extracted core features of the disease and summarized them in Table 1 and Table 2.

Frequencies are approximate and derived from patient-level data extracted from each source publication; they reflect the span of observed proportions across reports in which the relevant feature was systematically assessed and should not be interpreted as pooled epidemiological estimates. The categories “core feature”, “supportive feature”, and “emerging/expanded feature” represent the authors’ synthesis of the reviewed literature and are not based on pre-established diagnostic criteria. A core feature is defined here as one documented in ≥85% of assessable patients, a supportive feature as one present in 20–84% or reported in multiple independent publications, and an emerging/expanded feature as one described only in isolated cases. The conspicuous absence of rib anomalies, together with the presence of carpal/tarsal fusion and the lack of symphalangism, is repeatedly cited as the key radiological discriminator between SCT and other genetic vertebral segmentation defects (e.g., spondylocostal dysostosis, Klippel–Feil syndrome, multiple synostosis syndrome).

## 4. Discussion

This review consolidates clinical, radiological, and molecular data from eleven previously published reports of *FLNB*-related spondylocarpotarsal synostosis syndrome (SCT) and integrates them with a novel, molecularly confirmed case. Taken together, the reviewed cohort confirms that disproportionate short stature with a short trunk, contiguous vertebral fusion, and carpal synostosis—most typically involving the capitate and hamate—constitute the near-universal core triad of the syndrome, present in the large majority of reported patients (Table 2). Scoliosis and/or lordosis are similarly frequent, whereas tarsal fusion, hearing loss, facial dysmorphism, and dental anomalies are variably present and appear to depend on the age at assessment, the extensiveness of the diagnostic work-up, and possibly on locus- or variant-specific effects.

The novel case reported here broadens the recognized phenotypic and genotypic spectrum of *FLNB*-related SCT in several respects. First, the extent of axial involvement—with vertebral fusion and posterior arch clefts spanning the cervical, thoracic, and lumbosacral spine, together with coccygeal dysgenesis—appears more extensive than in most previously reported patients, in whom fusion is typically confined to a contiguous thoracic segment. Posterior arch clefts at multiple, non-contiguous levels and coccygeal involvement have not been systematically described in the reports reviewed here, suggesting that the axial phenotype of SCT may be more heterogeneous, and in some cases more severe, than previously appreciated. Second, our patient carries a novel homozygous frameshift variant, c.7332dup, predicted to introduce a premature stop codon (p.Met2445HisfsTer5) in the *FLNB* coding sequence; this adds to the growing catalogue of loss-of-function variants distributed across the gene (Supplementary Table S1) and is consistent with the mechanism common to essentially all molecularly confirmed SCT cases, namely biallelic loss of filamin B function through nonsense, frameshift, splicing, or large deletion variants, in contrast to the dominant-negative missense and in-frame variants that cause the clinically distinct, autosomal dominant *FLNB*-related disorders (Larsen syndrome, atelosteogenesis I/III, boomerang dysplasia).

The global developmental delay documented in our patient is more difficult to interpret in isolation. Most reviewed reports describe normal cognitive development in *FLNB*-related SCT, which is generally considered a primarily skeletal disorder without intrinsic neurodevelopmental impairment. In our case, however, prematurity, very low birth weight, early institutional care with limited developmental stimulation, and neuroimaging findings suggestive of possible perinatal hypoxic–ischemic injury, as well as other genetic causes not explored via targeted exome sequencing, represent plausible alternative or contributing explanations for the developmental delay, independent of the *FLNB* variant itself. This distinction is clinically relevant, as it should not be assumed that developmental delay is an intrinsic feature of SCT; rather, it underlines the importance of considering perinatal and environmental history when interpreting neurodevelopmental findings in adopted or internationally relocated children with a skeletal dysplasia diagnosis.

The absence of rib anomalies in our patient, despite extensive vertebral involvement, is consistent with the classical radiological definition of SCT and supports its distinction from spondylocostal dysostosis and other segmentation disorders, in which rib anomalies are a defining feature. This distinction remains one of the most consistent and clinically useful discriminators identified across the reviewed literature (Table 2), even though rare exceptions with secondary or atypical rib involvement have been reported and should not, by themselves, exclude the diagnosis.

The absence of tarsal synostosis at the time of the most recent assessment in our patient is in line with the considerable variability of this feature across the reviewed cohorts, where reported frequency ranges from 0% to 80% depending on the series and on the age at radiographic assessment. This variability suggests that tarsal fusion may be a late-onset or progressive finding in at least some patients, reinforcing the need for longitudinal radiological follow-up rather than a single cross-sectional assessment, since a negative finding at an early age does not exclude its later development. It is also worth noting that the absent navicular ossification center observed in our patient represents a distinct radiological finding from tarsal synostosis and most likely reflects the generalized delay in skeletal maturation already documented on hand radiographs, since ossification of the navicular is not typically expected before approximately 2–3 years of age. Nonetheless, as this specific finding has not been systematically reported among the reviewed *FLNB*-related SCT cohorts (Table 2), continued radiological follow-up will help clarify whether it reflects delayed ossification alone or an additional, previously unrecognized tarsal anomaly.

From a broader perspective, this review highlights several limitations inherent to the current *FLNB*-related SCT literature. All available evidence derives from case reports and small case series, with substantial heterogeneity in the completeness and standardization of clinical and radiological reporting; frequency estimates for individual features (Table 2) should therefore be interpreted as indicative rather than precise epidemiological figures. Publication bias toward more severe or diagnostically unusual presentations is also likely, which may inflate the apparent prevalence of certain findings. Furthermore, systematic genotype–phenotype correlation remains limited by the small number of molecularly confirmed cases; the majority of reported variants are predicted to cause a loss of function of the gene product, consistent with biallelic loss of filamin B function being the underlying disease mechanism, in keeping with the autosomal recessive inheritance of SCT.Larger, prospectively collected cohorts with standardized clinical and radiological assessment protocols, ideally coordinated through international rare disease registries, would help clarify the natural history of *FLNB*-related SCT, refine genotype–phenotype correlations, and better define which features should prompt specific surveillance (e.g., neurosurgical evaluation for craniocervical junction anomalies or audiologic monitoring), as opposed to those that can be managed expectantly. In the meantime, structured multidisciplinary follow-up, as outlined for our patient, remains the mainstay of management in the absence of a disease-specific treatment.

## 5. Conclusions

*FLNB*-related spondylocarpotarsal synostosis syndrome is an ultrarare, likely underdiagnosed autosomal recessive skeletal dysplasia characterized by disproportionate short stature, contiguous vertebral fusion, and carpal synostosis, with variable involvement of the tarsal bones, hearing, and craniofacial structures. Integration of data from eleven previously published reports (comprising 38 molecularly confirmed patients) with a novel case carrying a new homozygous frameshift *FLNB* variant confirms the core diagnostic triad while broadening the recognized phenotypic spectrum, in particular regarding the extent and multisegmental distribution of vertebral and posterior arch involvement. Early recognition of the radiological pattern—vertebral fusion without rib anomalies, combined with carpal synostosis—together with confirmatory molecular testing of *FLNB*, allows for a timely diagnosis and enables structured, multidisciplinary long-term surveillance, which currently represents the mainstay of management in the absence of a disease-specific treatment. Larger, prospectively collected cohorts are needed to refine genotype–phenotype correlations and to define evidence-based, disease-specific follow-up protocols.

## Figures and Tables

**Table 1:** Comparative summary of clinical and radiological key features across the eleven reviewed reports and the novel case.

		Key Clinical Features					Key Radiological Features			
Study	N Patients/Families	Short Stature	Short Neck	Scoliosis	Flat Feet/Club Feet	Hearing Loss	Multiple Vertebral Fusion	Carpal Fusion	Delayed Carpal Bone Age	Tarsal Fusion
Krakow et al. 2004 [10]	4/4	n/a	n/a	n/a	n/a	n/a	3/4	4/4	n/a	1/4
Mitter et al. 2008 [16]	1/1	1/1	1/1	1/1	1/1	1/1	1/1	1/1	1/1	n/a
Brunetti-Pierri et al. 2008 [13]	1/1	1/1	n/a	1/1	n/a	0/1	1/1	1/1	1/1	0/1
Yang et al. 2017 [9]	2/1	2/2	2/2	2/2	2/2	1/2	2/2	2/2	n/a	0/2
Salian et al. 2018 [6]	10/7	10/10	6/10	9/10	n/a	n/a	10/10	9/10	n/a	5/10
Yasin et al. 2021 [5]	5/1	5/5	5/5	3/5	1/5	2/5	5/5	4/5	n/a	3/5
Fukushima et al. 2021 [12]	2/2	2/2	n/a	1/2	1/2	2/2	2/2	2/2	n/a	1/2
Shahid et al. 2023 [4]	3/1	3/3	3/3	3/3	3/3	0/3	1/1	2/2	1/1	2/2
Ramos-Mejía et al. 2024 [3]	7/4	6/7	n/a	6/7	n/a	1/7	7/7	7/7	6/7	0/3
Qasim et al. 2024 [17]	1/1	1/1	n/a	n/a	1/1	n/a	1/1	n/a	n/a	n/a
Yousaf et al. 2026 [7]	2/1	2/2	2/2	2/2	2/2	2/2	2/2	1/2	n/a	1/2
Novel case report	1/1	1/1	1/1	1/1 *	1/1	0/1	1/1	1/1	1/1	0/1
**Total**	**39/25**	**34/35 (97%)**	**20/24 (83%)**	**29/34 (85%)**	**12/17 (70%)**	**9/24 (37%)**	**36/37 (97%)**	**34/37 (92%)**	**10/11 (91%)**	**13/32 (41%)**

* Scoliosis detected radiologically but not clinically evident on physical examination. n/a = not applicable.

**Table 2:** Aggregated frequency and diagnostic weight of clinical and radiological features of *FLNB*-related SCT across the reviewed literature and the novel case.

Clinical/Radiological Feature	Approx. Frequency Range Reported	Considered a Core Diagnostic Feature?	Representative Source(s)
Disproportionate short stature (short trunk)	~90–100%	Yes—core feature	All 12 reports
Vertebral fusion (contiguous, most often thoracic)	~90–100%	Yes—core feature	Krakow 2004 [10]; Salian 2018 [6]; Yang 2017 [9]; Novel case report
Scoliosis and/or lordosis	~80–92%	Yes—core feature	Yang 2017 [9]; Yasin 2021 [5]; Ramos-Mejía 2024 [3]; Novel case report
Carpal fusion (most commonly capitate–hamate)	~85–100%	Yes—core feature	Salian 2018 [6]; Ramos-Mejía 2024 [3]; Yasin 2021 [5]; Novel case report
Delayed carpal bone age/ossification	Frequently reported, not systematically quantified	Yes—supportive feature	Mitter 2008 [16]; Ramos-Mejía 2024 [3]; Yousaf 2026 [7]; Novel case report
Tarsal (foot) fusion	~0–80% (highly variable; often absent at first assessment)	Supportive but inconsistent	Ramos-Mejía 2024 (0/3) [3]; Salian 2018 (5/10) [6]
Short neck	~50–90%	Supportive feature	Yang 2017 [9]; Salian 2018 [6]; Shahid 2023 [4]; Novel case report
Clinodactyly of the 5th finger	Variable, commonly noted	Supportive feature	Yang 2017 [9]; Ramos-Mejía 2024 [3]
Joint mobility limitation (without fusion)	Variable	Supportive feature	Yasin 2021 [5]; Shahid 2023 [4]
Hearing loss (conductive, sensorineural, or mixed)	~20–60%	Supportive, not universal	Yang 2017 [9]; Yasin 2021 [5]; Fukushima 2021 [12]
Cleft/high-arched palate	Minority of cases (<25%)	Supportive, not universal	Reported in a minority across the reviewed *FLNB*-positive cohorts
Dental enamel hypoplasia	Minority of cases	Supportive, not universal	Brunetti-Pierri 2008 [13]; Mitter 2008 [16]
Facial dysmorphism (round face, broad nose, frontal bossing)	Variable; absent in a sizeable minority	Supportive, not universal	Ramos-Mejía 2024 (7/7) [3]; Salian 2018 (2/10 only) [6]; Novel case report
Flat feet/club feet	Variable	Supportive feature	Yasin 2021 [5]; Qasim 2024 (clubfoot) [17]
Rib anomalies (crowding, malalignment, fusion)	Rare—classically considered ABSENT in SCT	Used to EXCLUDE other vertebral segmentation disorders; rare exceptions reported	Yang 2017 (present, atypical) [9]; Salian 2018 (secondary crowding only) [6]
Epiphyseal dysplasia (e.g., proximal femur)	Rare, reported in isolated cases	Emerging/expanded feature	Mitter 2008 [16]
Normal intelligence/cognitive development	Reported as normal in the large majority of cases	Supportive (helps exclude broader syndromes)	All reports with developmental assessment. Novel case report: not assessable, given possible perinatal complications and prematurity
Mild short stature in heterozygous carriers	Occasional, isolated observations	Not established—possible incomplete penetrance	Yasin 2021 [5]; Mitter 2008 [16]

## Data Availability

All data are available upon request.

## Supplementary Materials

The following supporting information can be downloaded at https://www.mdpi.com/article/10.3390/genes17091054/s1, Supplementary Table S1: Biallelic FLNB variants reported to cause spondylocarpotarsal synostosis syndrome (SCT).

## Abbreviations

SCT	Spondylocarpotarsal synostosis syndrome
FLNB	Filamin B (gene)
WES	Whole exome sequencing
NGS	Next-generation sequencing
array-CGH	Array comparative genomic hybridization
ACMG	American College of Medical Genetics and Genomics
PVS1/PM2	ACMG evidence codes for variant classification
PRISMA	Preferred Reporting Items for Systematic Reviews and Meta-Analyses
MRI	Magnetic resonance imaging
CT	Computed tomography
GH	Growth hormone
ENT	Ear, nose and throat
CDC	Centers for Disease Control and Prevention (growth charts)
