# Supplementary material for: Filamin B (FLNB)-Related Spondylocarpotarsal Synostosis Syndrome: Systematic Literature Review and Novel Case Report
Source: Genes (Basel). 2026 Aug 31;17(9):1054. doi: 10.3390/genes17091054 (PMC13606696; doi:10.3390/genes17091054)
Supplement: Supplementary file 1 [file genes-17-01054-s001.zip › genes-4510484-supplementary.pdf]

## Supplementary Materials

**Table S1. Biallelic FLNB variants reported to cause spondylocarpotarsal synostosis syndrome (SCT).**

| Reference | Variant(s) | Predicted Protein Change(s) |
|---|---|---|
| Our case | Homozygous c.7332dup | Homozygous p.(Met2445HisfsTer5) |
| Yousaf et al. 2026 [7] | Homozygous c.3243C>A | Homozygous p.(Cys1081*) |
| Qasim et al. 2024 [17] | Homozygous c.209C>T | Homozygous p.(Pro70Leu) |
| Ramos-Mejía et al. 2024 [3] | Homozygous c.1128C>G | Homozygous p.(Tyr376*) |
| Ramos-Mejía et al. 2024 [3] | c.[1128C>G];[2911dup] | p.[(Tyr376*)];[(Ala971Glyfs*122)] |
| Ramos-Mejía et al. 2024 [3] | Homozygous c.1128C>G | Homozygous p.(Tyr376*) |
| Ramos-Mejía et al. 2024 [3] | c.[992dup];[1128C>G] | p.[(Leu332Profs*13)];[(Tyr376*)] |
| Shahid et al. 2023 [4] | Homozygous c.220C>T | Homozygous p.(Gln74*) |
| Fukushima et al. 2021 [12] | Homozygous c.1346-1372_1941+389del | Homozygous p.(Ala449Glyfs*26) |
| Fukushima et al. 2021 [12] | Homozygous c.3127-353_4223-1836del | Homozygous p.(Val1043Alafs*8) |
| Yasin et al. 2021 [5] | Homozygous c.2911dup | Homozygous p.(Ala971Glyfs*122) |
| Salian et al. 2018 [6] | Homozygous c.6317del | Homozygous p.(Pro2106Argfs*12) |
| Salian et al. 2018 [6] | Homozygous c.1493del | Homozygous p.(Glu498Glyfs*4) |
| Salian et al. 2018 [6] | Homozygous c.1243C>T | Homozygous p.(Arg415*) |
| Salian et al. 2018 [6] | Homozygous c.1204del | Homozygous p.(Val402Tyrfs*88) |
| Salian et al. 2018 [6] | Homozygous c.28G>T | Homozygous p.(Glu10*) |
| Salian et al. 2018 [6] | Homozygous c.1429delinsCT | Homozygous p.(Val477Leufs*9) |
| Salian et al. 2018 [6] | Homozygous c.1592dup | Homozygous p.(His532Thrfs*9) |
| Yang et al. 2017 [9] | Homozygous c.7621dup | Homozygous p.(Ser2542Leufs*82) |
| Mitter et al. 2008 [16] | Homozygous c.6010C>T | Homozygous p.(Arg2004*) |
| Brunetti-Pierri et al. 2008 [13] | Homozygous c.5548G>T | Homozygous p.(Gly1850*) |
| Krakow et al. 2004 [10] | Homozygous c.6408del | Homozygous p.(Ser2137Leufs*4) |
| Krakow et al. 2004 [10] | Homozygous c.1945C>T | Homozygous p.(Arg649*) |
| Krakow et al. 2004 [10] | Homozygous c.7029T>G | Homozygous p.(Tyr2343*) |
| Krakow et al. 2004 [10] | c.[2452C>T];[4819C>T] | p.[(Arg818*)];[(Arg1607*)] |

All variants are reported on the NM_001457.4 reference transcript. Apart from c.209C>T (missense), all listed variants are truncating variants, predicted to result in a truncated and/or absent filamin B protein, consistent with a loss-of-function disease mechanism.
